# Atomic Coordination Reflects Peptide Immunogenicity

**DOI:** 10.3389/fmolb.2015.00077

**Published:** 2016-01-11

**Authors:** Georgios S. E. Antipas, Anastasios E. Germenis

**Affiliations:** ^1^Division of Materials Technology, National Technical University of AthensAthens, Greece; ^2^Department of Molecular Medicine, Hellenic Pasteur InstituteAthens, Greece; ^3^Department of Immunology and Histocompatibility, School of Medicine, University of ThessalyLarissa, Greece

**Keywords:** pMHC-TCR interaction, atomic pair correlation, short range order, cumulative coordination, functional avidity, structure-function relationship

## Abstract

We demonstrated that the immunological identity of variant peptides may be accurately predicted on the basis of atomic coordination of both unprotonated and protonated tertiary structures, provided that the structure of the native peptide (index) is known. The metric which was discovered to account for this discrimination is the coordination difference between the variant and the index; we also showed that increasing coordination difference in respect to the index was correlated to a correspondingly weakening immunological outcome of the variant. Additionally, we established that this metric quickly seizes to operate beyond the peptide scale, e.g., within a coordination shell inclusive of atoms up to a distance of 7 Å away from the peptide or over the entire pMHC-TCR complex. Analysis of molecular orbital interactions for a range of formal charges further revealed that the N-terminus of the agonists was always able to sustain a stable ammonium (NH${}_{3}^{+}$) group which was consistently absent in antagonists. We deem that the presence of NH${}_{3}^{+}$ constitutes a secondary observable with a biological consequence, signifying a change in T cell activation. While our analysis of protonated structures relied on the quantum chemical relaxation of the H species, the results were consistent across a wide range of peptide charge and spin polarization conditions.

## Introduction

The original work on the characterization of the Class I Tax antigen along with three, artificially synthesized, of its variants—which were declared as biologically diverse while stereochemically similar (Ding et al., 1999)—set the stage for the quantification of the control exerted by peptide tertiary structure on the synapse formed by a peptide-Major Histocompatibility Complex (pMHC) from a T cell receptor (TCR). However, the passage of the next decades would reveal that the causal condition operating on the pMHC-TCR structure-function relationship would remain alarmingly elusive. This is largely due to the appreciable span of length scales—between three and four orders of magnitude—which separates the molecular level of the immune response from any plausible fundamental mechanism operating on the atomic or electronic level. Over the same period, a substantial amount of research was devoted to the phenomenology involved in the immune synapse (van der Merwe, 2001; van der Merwe and Davis, 2003), with particular emphasis on peptide anchorage inside the MHC (Agudelo and Patarroyo, 2010) as well as on the issue of docking of the TCR with the pMHC complex and its subsequent thermodynamic stability (Wan et al., 2005, 2008), both of which were discussed within the premise of binding energetics. However, predicting a biological outcome on the basis of p-MHC binding energy or pMHC-TCR free energy has never been proven as consistently possible.

Previously we suggested that biological function may not be predicted based on energetics (Antipas and Germenis, 2015a,b), as all bonded (and indeed, certain non-bonded) interactions occur within the first coordination shell of short range order; in the same work we argued that, due to the uniformity of bond lengths (which are directly correlated to bond energies) across the entirety of protein tertiary structure, binding energetics will tend to be degenerate. Here, we will exemplify this claim for the case of the Tax antigen (Tanaka et al., 2010)—a transcriptional regulatory protein of the human T-cell leukemia virus posing an attractive target for anti-cancer vaccine development (Sundaram et al., 2003) due to its critical role in HTLV-1-associated leukemogenesis (Kannagi et al., 1991; Elovaara et al., 1993; Pique et al., 2000)—and three of its artificially synthesized variants (Ding et al., 1999) by additionally showing that coordination carries the “signature” of peptide immunological identity. The direct relationship between coordination and peptide function will, furthermore, be shown to constitute a physical observable of biological function in both, unprotonated and protonated peptide tertiary structure.

## Materials and methods

### Peptides

The antigens used were the cognate HTLV-1 Tax peptide (LLFGYPVYV, PDB entry 1AO7) (index peptide), the weak agonist (or null peptide) V7R (LLFGYPRYV, PDB entry 1QSE), the weak antagonist Y8A (LLFGYPVAV, Protein data bank—PDB entry 1QSF) and the antagonist P6A (LLFGYAVYV, PDB entry 1QRN). All complexes were presented by HLA-A201 and were bound to the human A6TCR (Ding et al., 1999); furthermore, all complexes have been functionally characterized by cell assays as well as by kinetic and thermodynamic measurements (Ding et al., 1999). In alignment to previous work of ours (Antipas and Germenis, 2015a,b), the current analysis has been performed in the gas phase. The assumption of absence of water molecules from the cleft is reasonable on the basis of a reported entropic advantage (Schamel and Reth, 2007).

### Peptide protonation and the induction of polarization

Protein structures resolved by X-ray diffraction (XRD) are unprotonated as the H species is not detectable by X rays due to the low form factor of the hydrogen atom (Stojilovic, 2012). In principle, in-silico peptide protonation requires calculation of the interaction energies between pairs of charged residues and the average protonation of each residue must be determined from the electrostatic energies. For a characteristic Monte Carlo solution of the problem of residue protonation, the reader is referred to early work by Beroza et al. (1991). Here, peptide backbone and side chain bonds on PDB structures were saturated by hydrogen atoms, followed by ab initio relaxation of the H species. During H relaxation, the C, N and O species were kept immobile in their *in-vitro* crystallized positions. We also took into account the possibility of charge and spin on peptide structure by calculating the electronic structure for a range of allowed spin multiplicities up to the quintet state. A complete set of charge and spin polarization conditions is given in our precursor work (Antipas and Germenis, 2015a,b). All peptides were assumed to experience a neutral pH; furthermore, all protonated structures were initially zwitterionic and two different sets of ab initio calculations were carried out. In the first set, all peptides were fully protonated zwitterions. In the second set, the hydroxyl functional groups on the hydrophilic phenol rings were deprotonated.

### Ab initio calculations

All-electron, spin unrestricted DFT calculations were performed with the Amsterdam density functiona (ADF) program (te Velde et al., 2001; Amsterdam Density Functional (ADF) Program, 2014). Electron exchange and correlation was addressed by the BLYP (Becke, 1988; Lee et al., 1988) functional within the generalized gradient approximation (GGA). Single-electron wavefunctions were expanded using the TZ2P uncontracted Slater-type orbital (STO) basis set, (a triple-ζ basis set with two sets of polarization functions) for all atoms. Non-aufbau occupations were discarded.

### Calculation of pair correlation functions

Coordination refers to atomic structure and expresses the tendency of atoms to be surrounded by other atoms (the coordination number indicates the number of atoms surrounding a reference atom). The calculation of atomic coordination is based on the initial formulation of a histogram of interatomic interactions, via calculation of the distances between all atom pairs and the assignment of these distances to bins of a predefined size (e.g., 0.1 Å; choice of the most appropriate bin size is a matter of experimentation (Antipas and Germenis, 2015a,b,c,d) but does not affect the results if chosen to be sufficiently small). By convention, the ith bin is assigned all interatomic distances, R, within the range r < R < r + Δr, where *r* = i^*^Δ r and Δr is the bin size. For example, the 10th bin is assigned all interatomic distances 1.0 Å < R < 1.1 Å, the 11th bin includes all distances for which 1.1 Å < R < 1.2 Å, etc. The number of distances in each bin then represents the value of the histogram in that bin. If all atom species are considered the histogram is representative of the total coordination, whereas if calculations are restricted to specific atom species the histogram represents a *partial*. In the current study, both the total and all of the partial histograms were calculated for the peptide structures. Numerical manipulation of the histogram yields two important pair correlation functions which lead up to the atomic coordination number: the Pair Distribution Function (PDF), also symbolized as g(r), and the Radial Distribution Function (RDF), symbolized as R(r).

The PDF is a statistical representation of interatomic distances (Antipas et al., 2012). The PDF was calculated by initially constructing the histogram of interatomic distances in respect to the real space coordinate, r. Calculation of the histogram involved the partition of space into bins, with a bin size equal to 0.1 Å. The PDF is expressed as

(1) $$g(r)=\frac{1}{2\pi Nr^{2}\rho_{0}}\sum_{j\;=\;1}^{N}\sum_{i>j}^{N}\delta (r-r_{ij})$$

where *N* is the number of peptide atoms, δ is the Dirac delta function and ρ_0_ is the number density N/V, where V is the volume of the simulation box containing the peptide and r_ij_ is the distance between the ith and jth atoms. The RDF was then calculated as

(2) $$\text{R}(\text{r})\text{=4}\pi {\text{r}}^{\text{2}}\rho_{\text{0}}\text{g}(\text{r})$$

and integrated to estimate atomic coordination, n, of any atom within a spherical shell defined by interatomic distances r_1_ and r_2_ as follows

(3) $${\text{n}}_{{\text{r}}_{1}}^{{\text{r}}_{2}}=\int_{{\text{r}}_{1}}^{{\text{r}}_{2}}\text{R}(\text{r})\text{dr}=\text{4}\pi \rho_{0}\int_{{\text{r}}_{1}}^{{\text{r}}_{2}}\text{g}(\text{r}){\text{r}}^{2}\text{dr}$$

In the latter expression, the cumulative coordination for each peptide up to any value of interatomic distance r_2_ may also be computed by setting r_1_ equal to zero. Similarly, the running difference between any pair of such cumulative coordination integrals may be calculated either as total coordination (i.e., disregarding atom species) or as partial coordination (i.e., for selected atom species pairs). All calculations of PDF, RDF and coordination were performed with PRDF (PRDF, 2014; Antipas, 2015).

## Results

### Unprotonated peptide structures

**Figure 1:**
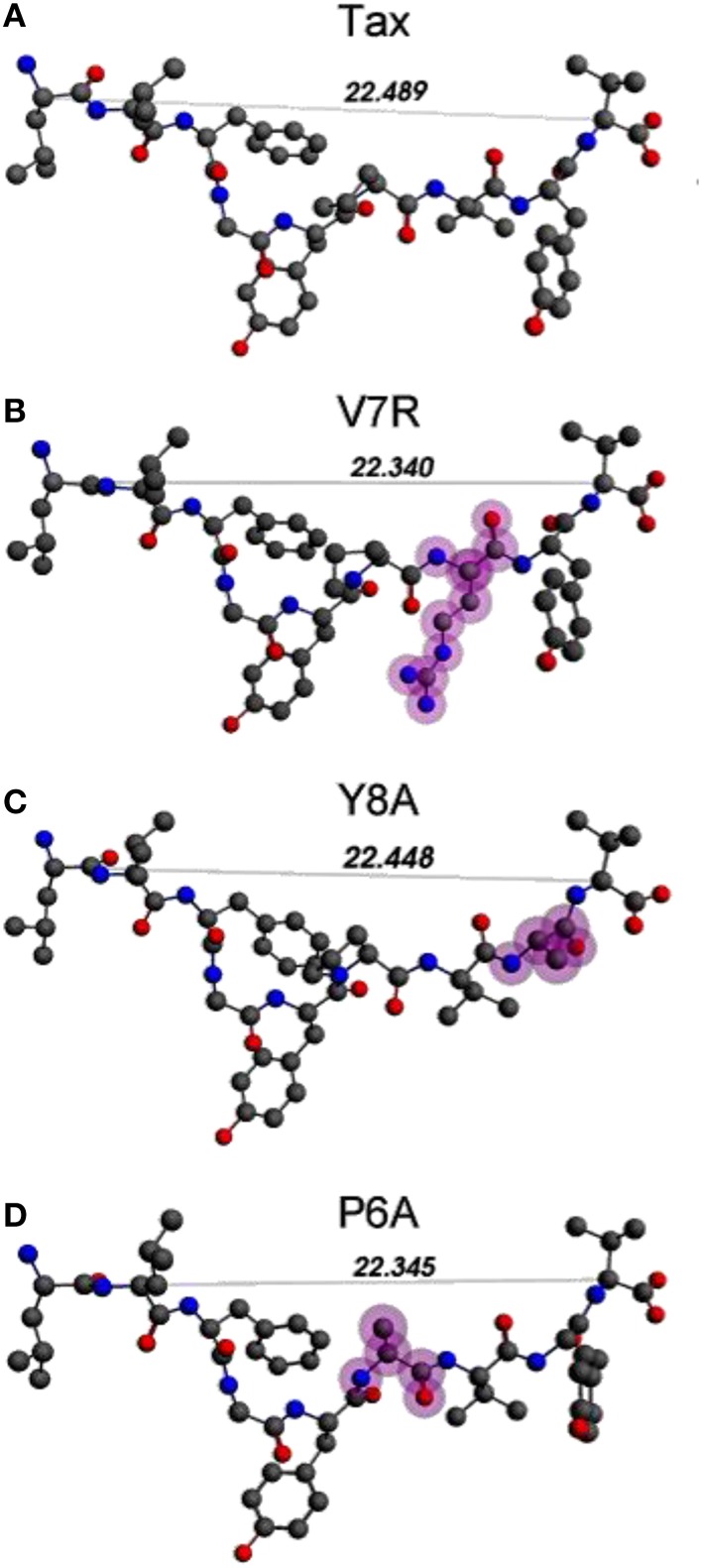
**(A–D)** Unprotonated structures of the Tax, V7R, Y8A, and P6A, respectively. The TCR alpha and beta chains (not shown) are located underneath the peptides while the MHC alpha chain (also not shown) is located over the peptide and encapsulates it. In all peptides, hydroxyl groups attached to the phenyl side chain of residue 5 point toward the alpha chain of the TCR. In every structure the distance in Å between the alpha carbon (C_α_) atoms of the N- and C-terminus residues are also shown. Atom color notation is C–gray, N–blue, and O–red and is followed throughout. Peptide mutations in respect to Tax are highlighted in purple.

Unprotonated peptide structures appeared similar, as judged by the distances between alpha carbon atoms (C_α_) on the N- and C-terminus residues (see Figure 1), all distances being of the order of 22 Å. Further comparison of the C_α_ distances revealed that antagonist, P6A, was marginally more confined than the agonist, Tax. The increased confinement of the P6A peptide was also reflected on its elevated density (equal to 0.309146 g/cm^3^, number density: 0.014444 atoms/Å^3^) as compared to that of Tax (density: 0.302837 g/cm^3^, number density: 0.014175 atoms/Å^3^). Also compared to Tax, the increased density of the weak agonist, V7R, (equal to 0.317370 g/cm^3^, number density: 0.014818 atoms/Å^3^), and the low density of the weak antagonist, Y8A, (density: 0.262836 g/cm^3^, number density: 0.012275: atoms/Å^3^) indicated respective over- and under-coordination, the latter probably reflecting more fundamental differences of backbone/sidechain conjugation (Li et al., 2009).

**Figure 2:**
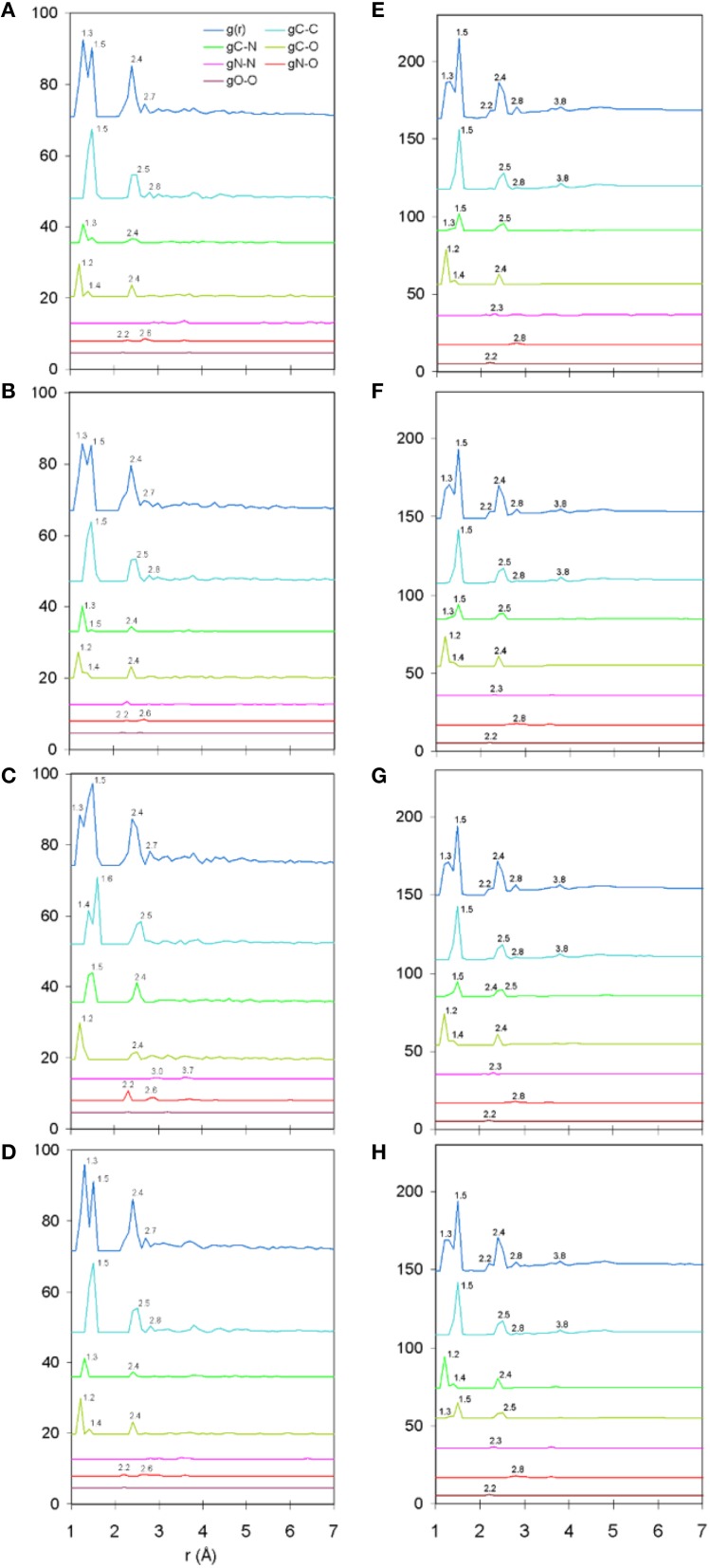
**Unprotonated structure total and partial PDF curves (symbolized as g(r) and gA-B, respectively where “A” and “B” are any of the C, N, or O species)**. From **(A–D)**: single peptides Tax, V7R, Y8A, and P6A, respectively. From **(E–H)**: entire pMHC-TCR complexes of the Tax, V7R, Y8A, and P6A peptides, respectively. All interatomic distances have been rounded up to the first decimal digit. Each of the partial curves has been normalized by the average number density of the peptide.

**Figure 3:**
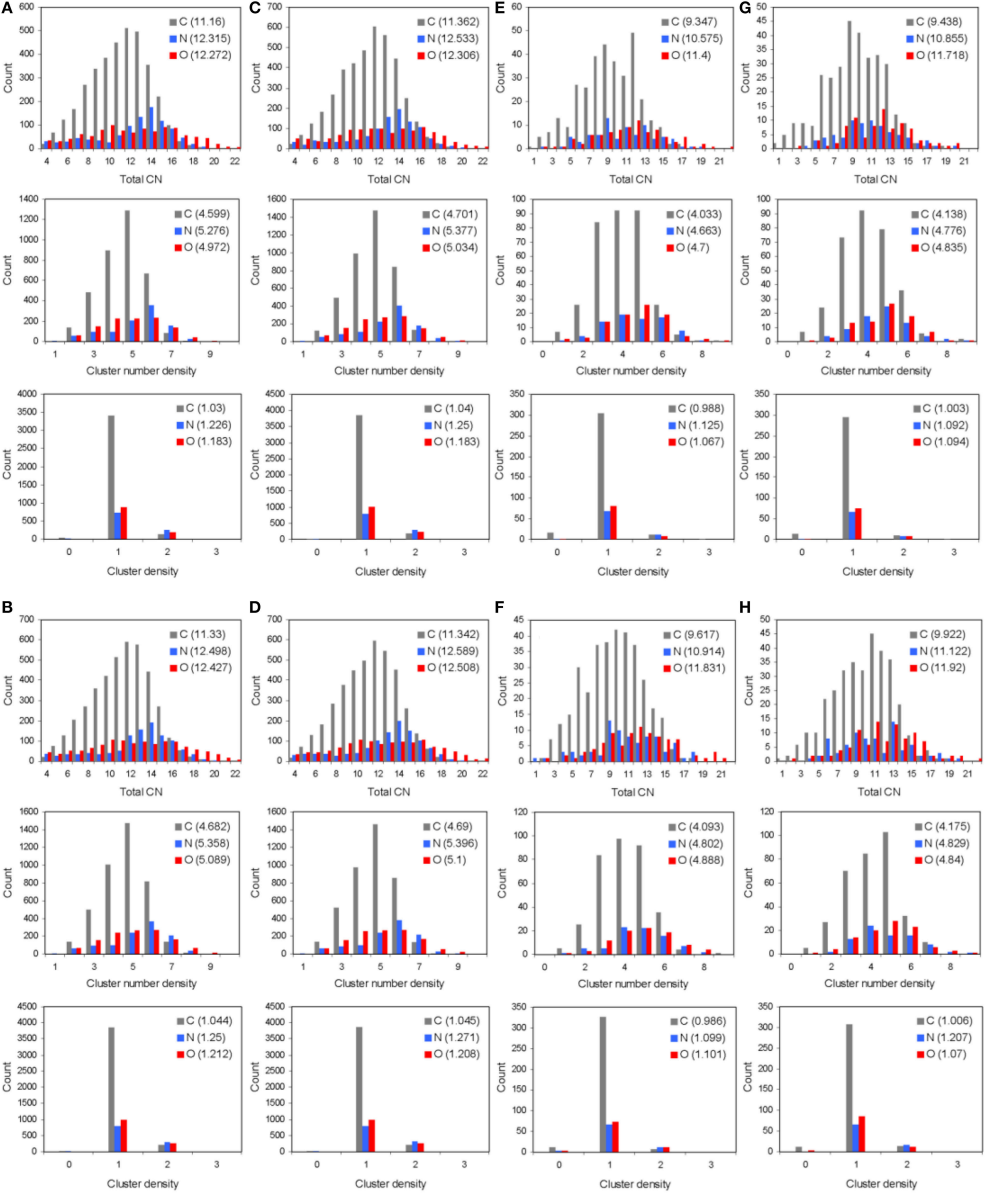
**Short range order statistics (total coordination number, CN, number density, and density, up to interatomic distances of 4 Å) for unprotonated pMHC-TCR complexes of (A) Tax, (B) V7R, (C) Y8A, and (D) P6A**. Also, short range order statistics for unprotonated complex environments including atoms up to a distance of 7 Å from peptide atoms: **(E)** Tax, **(F)** V7R, **(G)** Y8A, and **(H)** P6A. Statistics are shown for the C, N, and O species as differently colored bars. Weighted averages for each atom are shown in parentheses and are defined as (Σx_i_N_i_)/ΣN_i_, where x_i_ is the ith PDF bin and N_i_ is the histogram value.

The total and partial PDF of unprotonated peptide structures are shown in Figures 2A–D. The peak positions in every PDF curve underpinned our previously discussed theme of bond length uniformity within the first coordination shell (Antipas and Germenis, 2015a,b), the latter extending up to approximately 1.6 Å as indicated by the total PDF datasets. More precisely, the first coordination shell primarily comprised C-C and C-N bonded interactions, which were respectively 1.5 and 1.3 Å in length, regardless of the peptide (bond lengths are rounded up to a single decimal point). In fact, the consistency in the lengths of the C-C and C-N partials was characteristic of the entire pMHC-TCR complexes, as portrayed in Figures 2E–H. Short range order features were visible up to 4 Å, altogether faded beyond approximately 5–7 Å and this was common to the structures of both the isolated peptide and the entire pMHC-TCR complex. Within the realm of short range order, the average coordination, number density and density of atomic clusters centered on each of the C, N, and O species were also studied, with the aim of highlighting features which would be prominently indicative of agonist-antagonist discrimination. Two types of structures were considered: isolated peptides, the results for which are shown in Figures 3A–D, and the peptide inclusive of its cleft environment to within 7 Å from each peptide atom, shown in Figures 3E–H. The results indicated that average cluster coordination, density and number density were always higher on the variants in comparison to Tax; therefore peptide biological function may not be readily discerned on the basis of the first coordination shell. Perhaps surprisingly, the cleft environment to within 7 Å from peptide atoms was also found to be devoid of a trend signifying a clear structure-function relationship.

**Figure 4:**
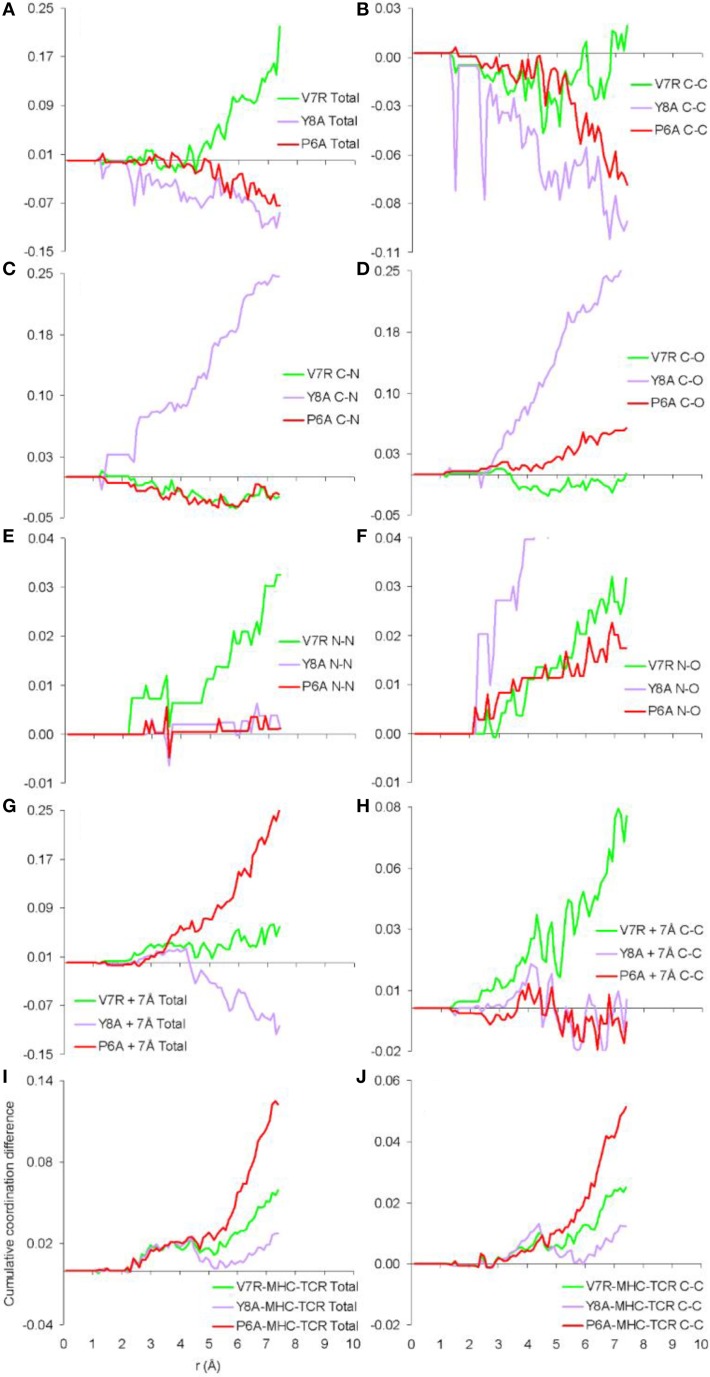
**Cumulative coordination differences of unprotonated structures in respect to Tax**. From **(A–F)** single peptides, **(G,H)** peptides and a surrounding shell inclusive of atoms up to 7 Å from each of the peptide atoms and **(I,J)** the entire pMHC-TCR complexes.

Previously we established that cumulative coordination difference of the protonated variant peptides in respect to the index is correlated to immunological identity (Antipas and Germenis, 2015a,b,c,d) and, more precisely, that cumulative under-coordination is characteristic of the antagonist; at this point we inquire if this might additionally hold true for unprotonated structures and the results are shown in Figure 4, for the cases of the isolated peptides, the cleft environment up to 7 Å from each peptide and the entire pMHC-TCR complex. Both the total PDF (Figure 4A) and the C-C partial (Figure 4B) were found to be correlated to peptide immunological identity (i.e., antagonist peptides were under-coordinated in respect to Tax), in agreement with our previous results of protonated structures; in contrast, he rest of the PDF partials (Figures 4C–F) were not relevant. Furthermore, the total PDF was not found to reflect function either on the scale of the immediate environment of the peptide (see Figures 4G,H) or in the premise of the entire pMHC-TCR complex (see Figures 4I,J). Therefore, we envisage that the physical observable of pMHC-TCR functional avidity is closely associated with the scale of the peptide.

### Peptide protonation

**Figure 5:**
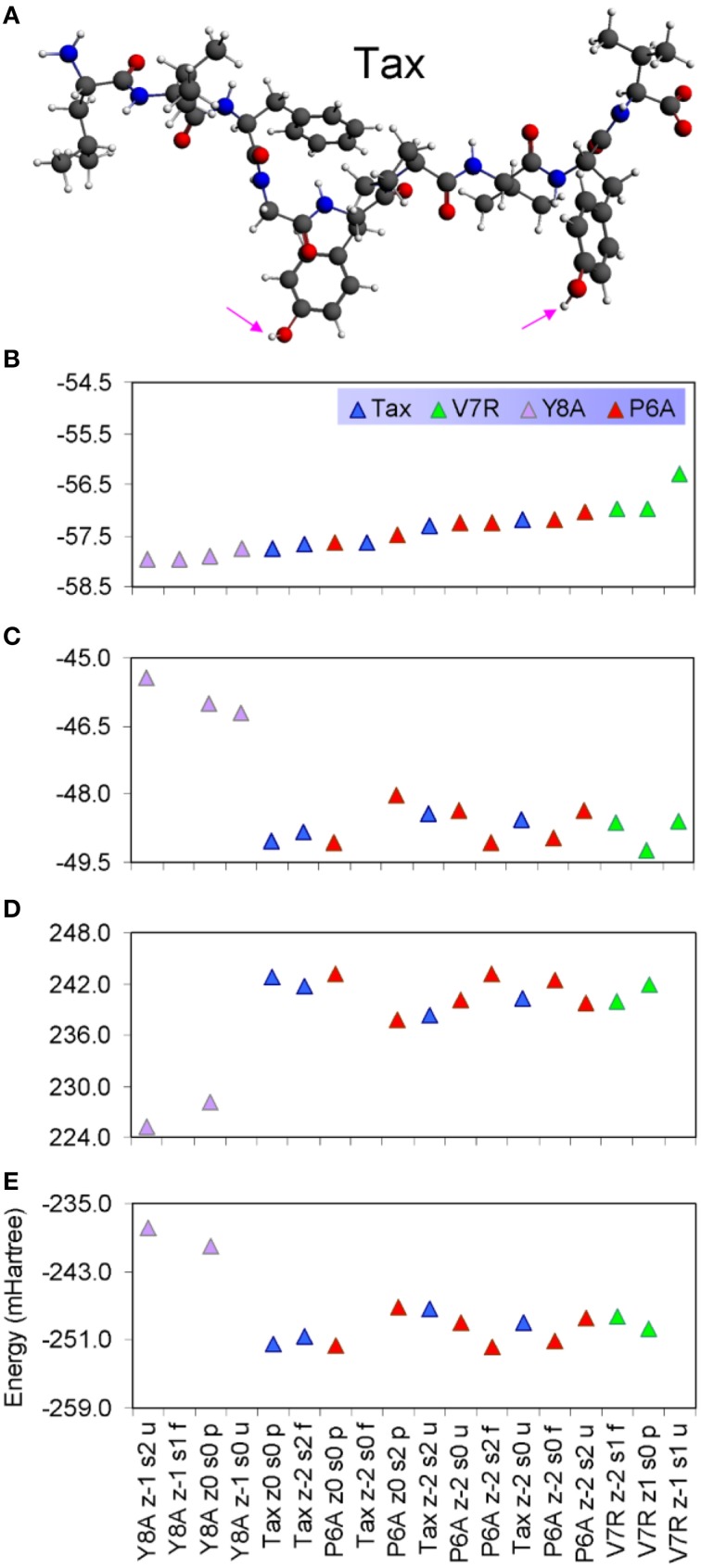
**(A)** The protonated Tax structure in which H atoms are shown in white color. We deemed that each of the protonated peptides could potentially give rise to two stereochemical variants: one in which the hydroxyl groups attached to phenol side chains would be fully protonated and one in which the hydroxyls would be deprotonated; these stereochemical variants are referred to as “p” and “u” respectively throughout the text. **(B)** Total binding energies of a range of protonated peptides, sorted in ascending energy order. The decomposition of binding energy into its constituent electrostatic, Pauli repulsion and orbital interaction parts is shown in **(C–E)**, respectively.

Protonated tertiary structures were subjected to various deprotonation combinations of their hydrophilic side chain hydroxyl groups (Antipas and Germenis, 2015b; see the groups marked by cyan arrows in Figure 5A), in order to cater for the possibility of excessive alkalinity experienced by the side chain in the TCR micro environment. The compounded effect of the different deprotonation/charge/spin combinations as reflected on the binding energy of the peptides is shown in Figures 5B–E. On the basis of total binding energy (Figure 5B), the models of the weakly interacting variants (Y8A and V7R) were observed to lie in the lowest and highest energy positions, respectively. Unfortunately, between the two energy extremes, there was pronounced overlap among the binding energies of the agonist and the antagonist. Moreover, the energy constituents (electrostatic, Pauli and orbital, shown in Figures 5C–E respectively) did not reveal a solid trend in regard to peptide functionality and, in fact, confirmed that the binding energies of the agonist and antagonist models were degenerate; due to these factors peptide binding energy was rendered unsuitable as a criterion of functional avidity. We, thus, proceeded to examine the pair distribution function of peptide protonated structures.

**Figure 6:**
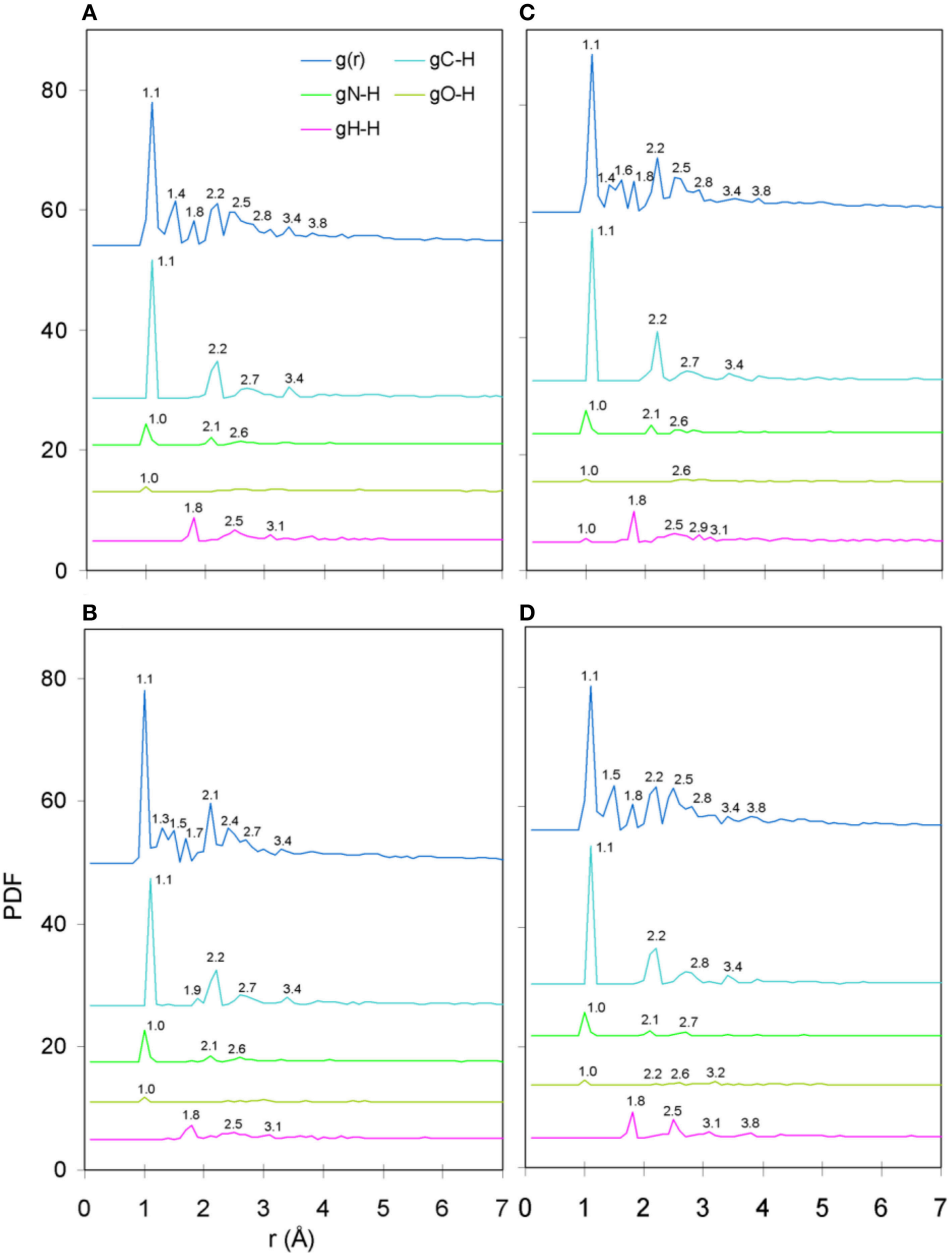
**PDF diagrams of the H species partials for protonated, spin unpolarized peptide models: (A) Tax, (B) V7R, (C) Y8A, and (D) P6A**.

**Figure 7:**
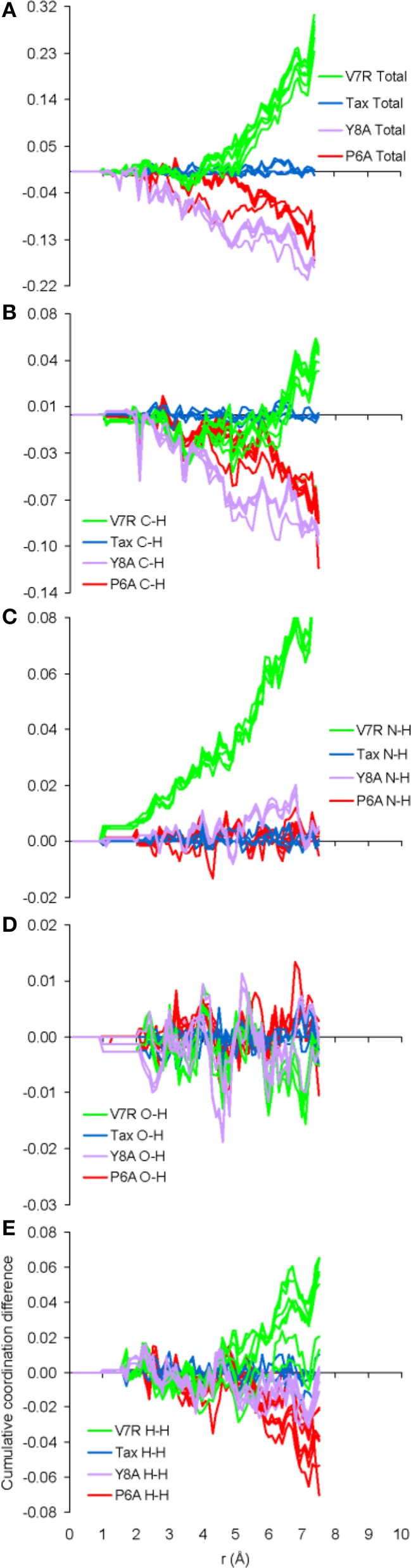
**Cumulative coordination difference of the partials of the H species for peptide protonated structures in respect to the charge-neutral spin-unpolarized Tax model**. From **(A–E)** the total and C-H, N-H, O-H, and H-H partials, respectively.

The only meaningful differences between the PDF curves of the unprotonated and protonated tertiary structures comprised partials of the H species and these are shown in Figure 6, for the case of spin unpolarized peptides. Principal contributions to the total PDF were from the C-H partial, which also exhibited the most varied behavior in respect to peaks beyond the first coordination shell; more specifically, V7R (Figure 6B) was the only structure showing a peak at 1.9 Å while P6A (Figure 6D) exhibited a peak at 2.8 instead of 2.7 Å shown by the rest of the structures, a sign of lower second-shell coordination (longer interatomic distances). Aside from the total PDF of the unprotonated peptides and their C-C partial, the protonated structures have also been shown to reflect pMHC-TCR functional avidity (Antipas and Germenis, 2015a,b) through the total PDF (also see Figure 7A). Our current results indicate that primarily the C-H (Figure 7B) and secondarily the H-H partials were also correlated to functional avidity, while the rest of the H partials were not (see Figures 7C–E).

### Contribution of residue interactions toward coordination differences

**Figure 8:**
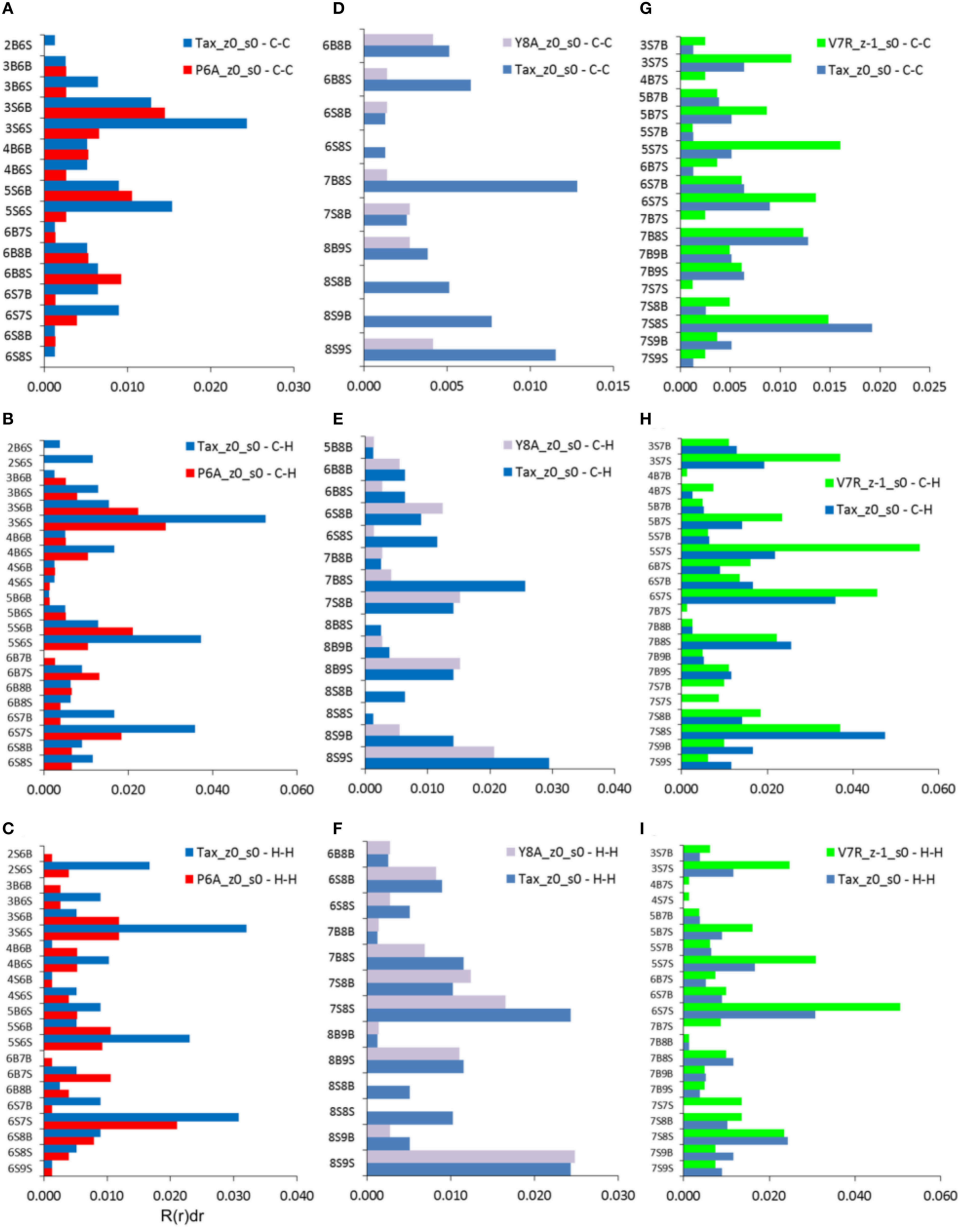
**Coordination, R(r)dr, per type of interaction (vertical axes) for the C-C, C-H, and H-H partials of non-polarized peptide structures**. All graphs include contributions by Tax for the sake of comparison: **(A–C)** P6A, **(D–F)** Y8A, and **(G–I)** V7R. All data refer to interatomic distances within the range 5–7 Å. Each interaction represents a group of interatomic distances and is symbolized by the number of the residue hosting each atom and a designation denoting a backbone, “B,” or side chain, “S,” atom position. For example, interaction “2B6S” denotes the coordination sum which arises from all interatomic distances between backbone atoms (“B”) on residue 2 and side chain atoms (“S”) on residue 6.

As already indicated by the total PDFs of both unprotonated and protonated peptide structures (see Figures 4A, 7A), coordination deviations in respect to Tax were correlated to peptide immunological identity at interatomic distances beyond 5 Å and up to the limit of short range order, at approximately 7 Å. Within this range we proceeded to calculate the coordination, R(r)dr, between backbone and side chain atoms for each of the three partials which were most actively implicated in peptide functionality, i.e., C-C, C-H, and H-H. The results indicated that a very specific subset of residue interactions was always responsible for coordination differences and we, thus, present in Figure 8 the case of non-polarized peptides without loss of generality. As may be deduced by comparison of Figures 8A,D,G, by far the highest contributors to peptide coordination deviations from Tax were the side chain atoms of the substituted residues, i.e., residues 6, 7, and 8 for P6A, V7R, and Y8A respectively. P6A under-coordination was underpinned by C-C interactions contributed solely by residue 6 side chain atoms (6S) at a substantial 81%; the corresponding C-C percentages for Y8A and V7R were 77 and 64%, respectively. Of equal importance were coordination deviation contributions by side chain C-H partials on substituted residues. These were 85, 73, and 69% for P6A, Y8A, and V7R, respectively. In some contrast, the H-H partial contributions were not as important, since the Y8A 8S contribution (see Figure 8I) was only 39% toward the peptide's total under-coordination in respect to Tax (these contributions for P6A and V7R were high, at 79 and 69% respectively).

### Quantum descriptors

**Figure 9:**
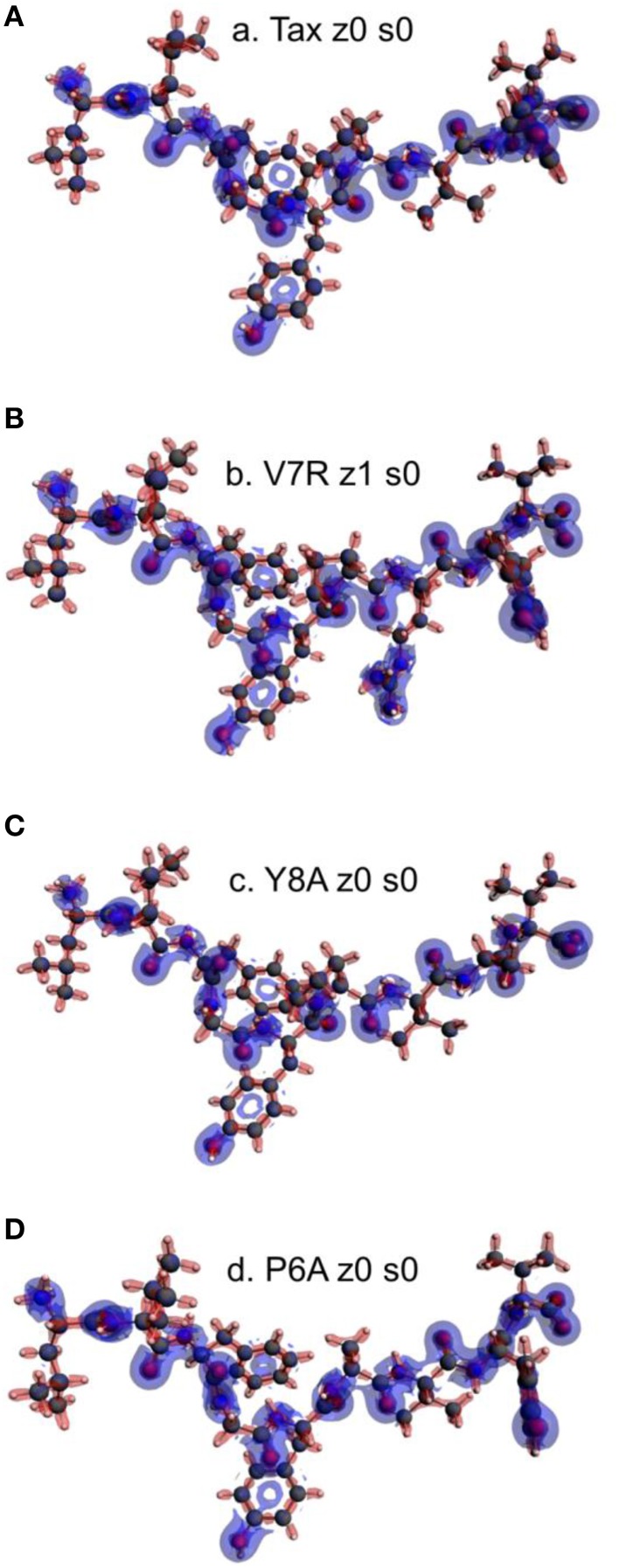
**Laplacian of the electron density of non-polarized peptide structures**. All triggering side chains are fully protonated. From **(A–D)**: Tax z0 s0, V7R z1 s0, Y8A z0 s0, and P6A z0 s0. Charge concentration (high potential energy) and charge depletion (high kinetic energy) are shown as red and blue surfaces, respectively. All iso-density surfaces are drawn at 0.2 a.u.

It is reasonable to expect that there may be potential for relationships to be drawn between the topology of the charge density and coordination. Accordingly, the Laplacian of the electron density for non-polarized peptide structures, presented in Figure 9, revealed a rather counter-intuitive similarity among agonists and antagonists; more specifically, it appeared that all peptide backbones and triggering side chains were loci of high electron kinetic energy (charge depletion) while hydrophobic side chains remained wrapped within regions of high potential energy (charge concentration). This uniformity of motifs across peptides did not allow any consistent conclusions to be drawn in regard to peptide functionality; hence a spectrum of formal charge and spin polarization combinations were introduced on the tertiary peptide structures, with the aim of detecting structural and/or electronic motifs consistent with immunological function. This analysis also considered the possibility of deprotonation of the terminal (hydroxyl) groups residing on the hydrophilic side chains.

**Figure 10:**
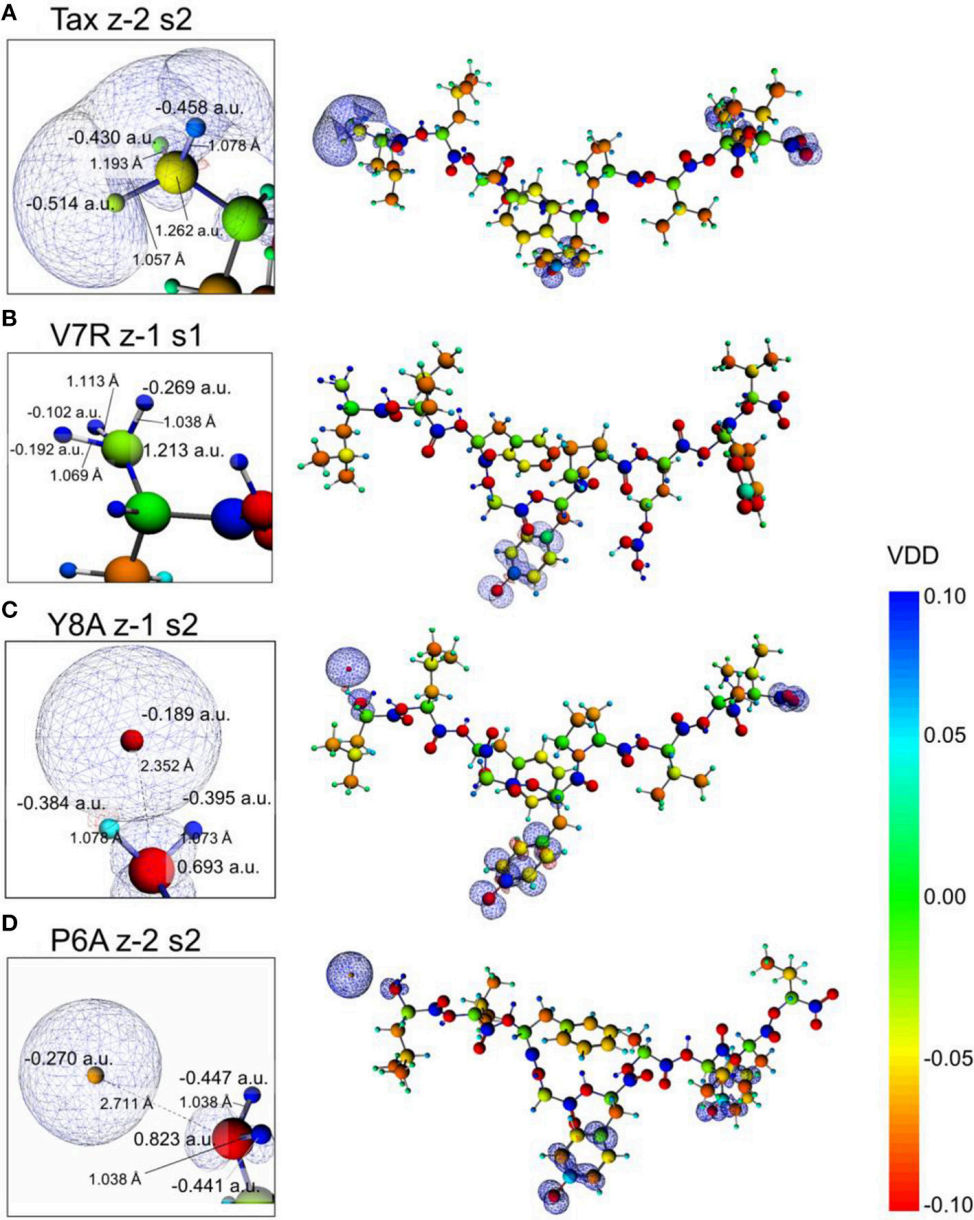
**Voronoi deformation density (VDD) and spin density of selected spin polarized peptides**. From **(A–D)**: Tax (charge -2, polarization 2), V7R, Y8A, and P6A (charge -2, polarization 2). All peptides had a formal charge of -2 and a spin polarization of 2 except V7R for which these quantities were -1 and 1, respectively. On all structures the terminal hydroxyl groups of the triggering side chains were deprotonated. Every inset depicts a magnification of the peptide *N* terminus, which shows atom Voronoi charges and bond lengths. All spin density surfaces are drawn at 0.002 a.u. The VDD bar values are also in a.u.

**Figure 11:**
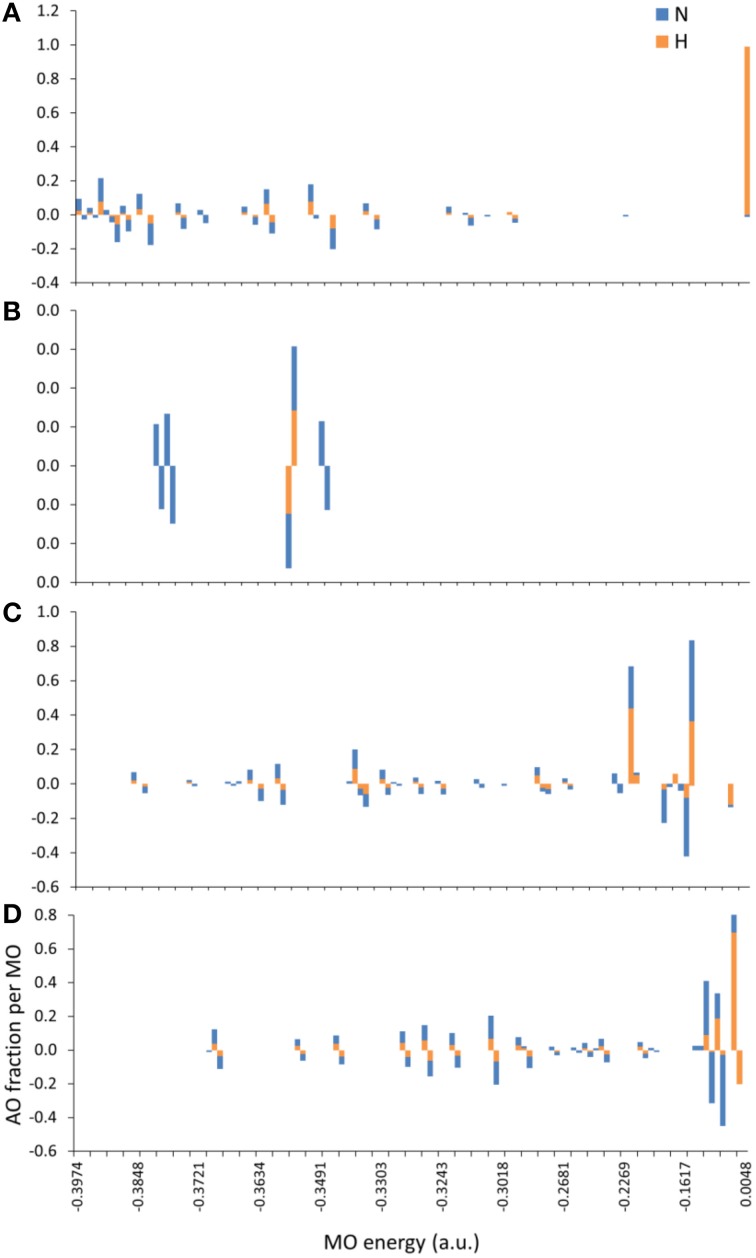
**MO contributions (fractions) by *N* terminus atomic orbital (AO) populations**. From **(A–D)**: Tax (charge (z) -2, spin polarization (s) 2), V7R (z-1, s1), Y8A (z-1, s2), and P6A (z-2, s2) respectively. All triggering side chains were deprotonated. Each bar corresponds to the sum of a_1g_ and t_1u_ contributions either by the *N* atom or by the group of the three H atoms on the *N* terminus. Majority (spin up) and minority (spin down) populations are shown as positive and negative AO fractions, respectively.

The introduction of spin polarization produced a very consistent difference in the structures of the agonist vs. that of the agonist peptides: all agonists maintained a zwitterionic state while antagonists did not and this behavior was observed across all the charge/spin combinations studied. A select such case is presented in Figure 10, in which we compare peptides of similar charges and spin polarizations. Regardless of their functionality, all peptides redistributed electron density such that there was depletion over backbone atoms and concentration over side chain (mostly O species) atoms as shown by the Voronoi deformation density (VDD) topology. However, the topology of spin density (shown as iso-density surfaces in Figure 10) was decidedly different among peptides of different functionalities and was accompanied by formation of an ammonium (NH${}_{3}^{+}$) group particular only to the Tax and V7R agonists. Although spin density was delocalized over all triggering side chains, it was manifested as a lobe encapsulating the *N* terminus H atoms of the Tax peptide (see Figure 10A), while it always engulfed the H atom separated from the *N* terminus of the antagonist structures (see Figures 10C,D). However, spin density was not delocalized over the *N* terminus of weak antagonist V7R. *N* terminus atomic orbital (AO) (sum of a_1g_ and t_1u_) contributions toward formation of molecular orbitals are shown in Figure 11. Again, our outlook for the analysis of AO contributions across the four peptides was qualitative; the data raised two features of interest. The first was that both antagonist peptides (Figures 11C,D) comprised valence molecular orbitals (MO) primarily made up by majority (spin up) H and minority (spin down) contributions toward formation of the highest-lying frontier orbitals (i.e., Highest Occupied Molecular Orbital—HOMO, as well as HOMO-1). The second, and perhaps most prominent, feature was that the native peptide, Tax, contributed its *N* terminus H density almost exclusively toward formation of the structure's low-lying Lowest Occupied Molecular Orbital (LUMO)—see H contribution at 0.0048 a.u. in Figure 11A; this was in direct contrast to the three other peptides compared and was consistent across all spin polarized models examined in the case of Tax.

## Discussion

PDF fluctuations gradually tailed off with increasing values of the real space coordinate, r. In practice, no appreciable structural features existed beyond 5 Å and were certainly absent beyond 7 Å (see Figure 2 for unprotonated and Figure 6 for protonated structure PDF's). Also, from the RDF expression (see Equation 2) it may be seen that the term *r*^2^ will naturally assign greater weight onto larger interatomic spacings; hence the RDF suggested that any interatomic interactions that are to be instrumental for peptide coordination are to be found in the range of 5 to 7 Å (broadly speaking, 5 Å is the scale of single residues). In the case of the peptide environment inclusive of structure within a radius of 7 Å from peptide atoms (see Figure 3E through to Figure 3H), coordination differences among peptides ranged up to 5% (between the Tax and P6A) while in the case of the entire complexes (Figure 3A through to Figure 3D) these differences were negligible. Accordingly, we are inclined to propose that pMHC-TCR functional avidity is not reflected on scales beyond that of the peptide. Moreover, the energetics involved in coordination did not present a singularly meaningful trend even on the scale of single peptides, as seen by the overlapping binding energies shown in Figure 5B. The extensive similarity among partials over scales ranging from the peptide up to the pMHC-TCR complex, shown in Figure 2, should be considered as supportive of the theme of first coordination shell energy degeneracy, raised by us previously (Antipas and Germenis, 2015b).

One of the most important findings of the current work was the correlation between the total PDF of unprotonated tertiary structures and pMHC-TCR functional avidity. This relation, which was expressed by peptide cumulative coordination differences from the index, is depicted in Figure 4. Coordination based on the total PDF was underpinned primarily by the C-C partial (Figure 4B) with contributions from the N-N partial (Figure 4E), the latter being particularly relevant to the weak agonist, V7R. Cumulative coordination based on the total PDF of unprotonated structures (the utility of the latter has been raised in our previous work, Antipas and Germenis, 2015a,b) was in full agreement with coordination based on the protonated models (Figure 7A). Interestingly, coordination differences within the short range limit of the peptides (i.e. up to a radial distance of 7 Å) were in agreement with functional avidity measurements (see Figure 4H) but they seized to be of relevance on the scale of the entire pMHC-TCR complex (Figures 4I,J). As already mentioned, along with C-C, both of the C-H and H-H partials (Figures 7B,E respectively) were correlated to peptide functionality and this correlation was controlled by the conformation of side chains on the substituted residues. It is notable that, in the case of the agonists, P6A and V7R, the side chains of the substituted residues were hydrophobic (i.e., buried in the MHC). Since coordination deviations arise mainly from hydrophobic side chains, sufficient rigidity of hydrophobic portions (Gakamsky et al., 2004; Schamel and Reth, 2007; Antipas and Germenis, 2015b) would favor the possibility that peptide agonistic potential is inborn to the pMHC complex upon its conformation and its presentation on the surface of the antigen presenting cell and, hence, independent of the TCR.

## Conclusions

The main points established to determine the link between selective agonist coordination and peptide immunological identity are:

1. The structural expression of agonism occurs over interatomic distances within the range of 5–7 Å, i.e., beyond the typical length of the residue; we therefore nominate this length as the interaction limit below which peptide immunological identity may not be defined. Any bonded interaction occurring over interatomic distances smaller than this critical scale will be reflective of “coordination shell degeneracy,” a term we introduced to describe a disconnection of the correlation between peptide immunological identity and structure.
2. It is unclear to which degree peptide tertiary structure is shaped by structural adaptations made by both the pMHC and the TCR, during formation of the immune synapse. However, as most of the peptide tertiary structure is hydrophobic, the correlation of immunological identity to interatomic distances beyond a critical 5–7 Å suggests that the structural expression of immunological identity (i.e., selective agonist coordination or the absence of it) must be in place already upon presentation of the immunocompetent peptide by the MHC, on the provision that the MHC cleft does not undergo structural adaptations larger than 5–7 Å during antigen recognition by the TCR.
3. Weak agonist and weak antagonist peptides are, respectively, markedly over- and under-coordinated in comparison to the native peptide. Hence the coordination requirement for an antagonist peptide is, indeed, very close to that of the agonist, albeit the antagonist is most categorically under-coordinated. This selective agonist coordination was found to be reflected upon the C-C, C-H, and H-H PDF partials.
4. Despite other structural similarities, the electronic structure of an agonist vs. that of an antagonist peptide is profoundly different. We determined that selective agonist coordination could be explained on the basis of specific charge and spin polarization combinations acting on peptide electronic structure in the gas phase. In the premise of polarization, antagonists were always found to maintain a stable ammonium group on their N-termini, which was altogether inaccessible to antagonists. Moreover, the density of states revealed that the index (Tax) contributed the bulk of the electron density associated with the H species of its ammonium group toward the peptide's LUMO.

## Author contributions

GA designed and performed the analysis and wrote the main body of the paper. Both authors discussed the results and commented on the manuscript at all stages.

### Conflict of interest statement

The authors declare that the research was conducted in the absence of any commercial or financial relationships that could be construed as a potential conflict of interest.
